# Supplementary material for: Slab morphology and deformation beneath Izu-Bonin
Source: Nat Commun. 2019 Mar 21;10:1310. doi: 10.1038/s41467-019-09279-7 (PMC6428881; doi:10.1038/s41467-019-09279-7)
Supplement: Supplementary file 1 — Supplementary Information [file 41467_2019_9279_MOESM1_ESM.pdf]

Supplementary Information to

Slab morphology and deformation beneath Izu-Bonin

Haijiang Zhang[1,2*], Fan Wang[1#], Robert Myhill[3*], Hao Guo[1]

[1]School of Earth and Space Sciences; Laboratory of Seismology and Physics of Earth's Interior, University of Science and Technology of China, 96 Jinzhai Road, Hefei, Anhui 230026, China

[2] CAS Center for Excellence in Comparative Planetology, 96 Jinzhai Road, Hefei, Anhui 230026, China

[3] School of Earth Sciences, University of Bristol, Queens Road, Bristol, BS8 1QU, UK

Now at Michigan State University, Department of Earth and Environmental Sciences, Natural Science Building, East Lansing, Michigan 48824, USA

* Correspondence and requests for materials should be addressed to zhang11@ustc.edu.cn (HZ) or bob.myhill@bristol.ac.uk (RM)

Supplementary Note 1 Teleseismic double-difference tomography of the Izu-Bonin subduction zone

In this study, we assembled arrival-time data from the International Seismological Center (ISC) dataset, as reprocessed by the single event location method of Engdahl, van der Hilst and Buland (EHB; Engdahl et al.[1]). Supplementary Figure 1 shows the distribution of global seismic stations used in this study. It is obvious that the original EHB catalog data contains some outliers from the distribution of travel times versus epicentral distance (Supplementary Figure 2). After removing the outliers outside the main trend of travel time curves in the EHB catalog, 893,359 absolute P wave arrival times are selected for the 9998 earthquakes in the Izu-Bonin region (Figure 1) recorded by global stations in the period of 1960 to 2008. The 2015 Mw7.9 Bonin earthquake (11:23:02 UTC) and its aftershocks are also included in the catalogue. From these absolute arrival times, a total of 4642513 P wave differential arrival times are constructed by choosing event pairs having more than 8 neighboring events and 8 observations. We require that the offset for event pair is at least 2 km when constructing differential arrival times. For the assembled differential arrival times, the average offset for all the event pairs is 54.7 km and the maximum offset is 299.97 km. Generally, the average offset should be close to and greater than the minimum inversion grid interval which in this case is 30 km in longitude.

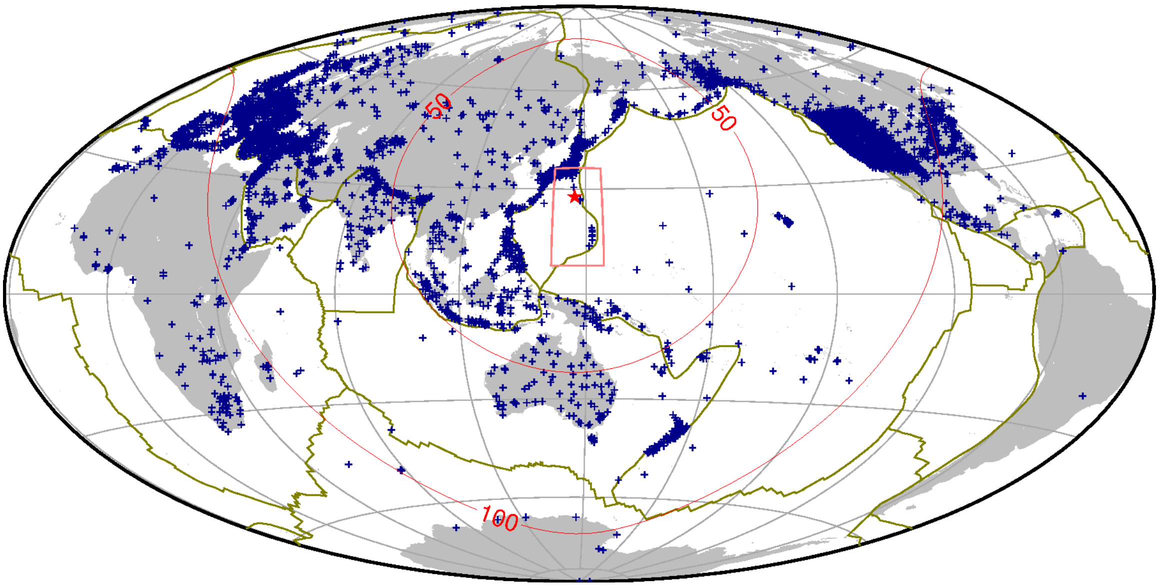

Supplementary Figure 1: Distribution of global seismic stations (blue pluses) used in this study. Epicentral distances of 50° and 100° are shown in red ellipses. The red star shows the location of the 2015 Bonin earthquake.

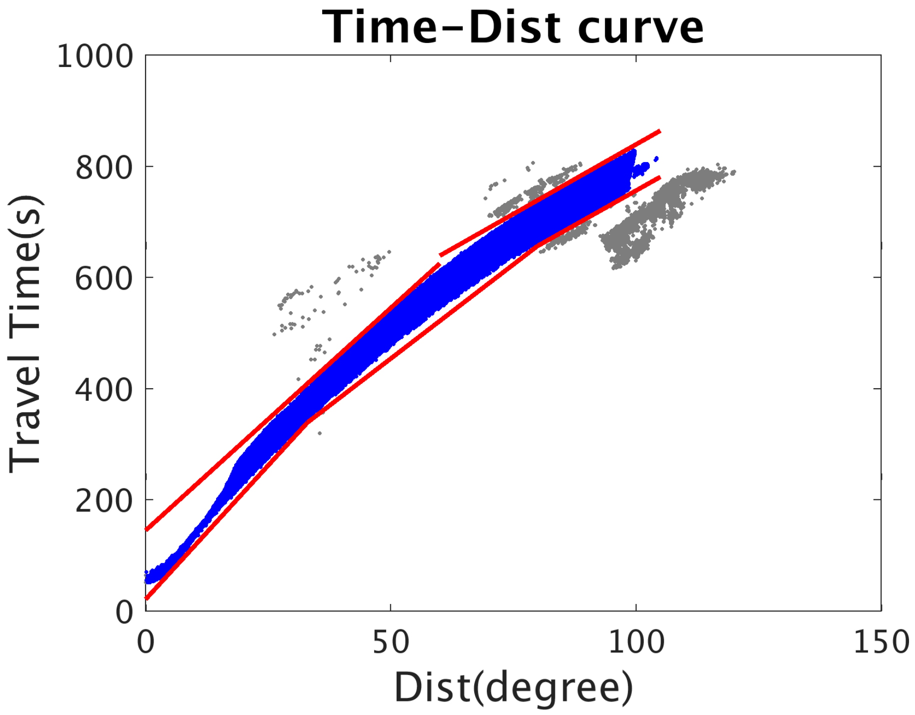

Supplementary Figure 2: Travel time curves for the arrival times assembled in this study. The blue dots are the data selected in this study and the outliers are plotted in gray. The red lines are used to distinguish between them.

The regional model in the tomographic inversion is bounded by the 8º N and 36º N lines of latitude, 134º E and 149º E lines of longitude, and extends from the surface to 900 km depth (Supplementary Figure 3). We adopted a two-step strategy for tomographic inversion, in which a coarser inversion grid is first used and the resulting velocity model is used as the starting model for an inversion using a finer inversion grid. In this way, the final inverted velocity model contains both large-scale and small-scale features. For this study, the inversion grid nodes in longitude and depth are fixed but in latitude we first use coarser grid and then finer grid. In depth, grid nodes are set at 0, 80, 150, 210, 270, 320, 370, 410, 440, 480, 520, 560, 600, 630, 660, 690, 740, 810, and 900 km (Supplementary Figure 3). In longitude, the inversion grid intervals are 0.3° where the 2015 Bonin earthquake happened but are larger near the edge of the regional model. The grid nodes in longitude are 134.0º, 135.2º, 136.2º, 137.0º, 137.6º, 138.1º, 138.5º, 138.9º, 139.2º, 139.5º, 139.8º, 140.2º, 140.5º, 141.0º, 141.7º, 142.5º, 143.4º, 144.5º, 145.8º, 147.3º, and 149.0º E (Supplementary Figure 3). In latitude, the grid nodes for the coarser grid are set with an interval of 3°-4° and are located at 8.0º, 12.0º, 16.0º, 20.0º, 23.5º, 27.0º, 30.0º, 33.0º, and 36.0º N (Supplementary Figure 3). The finer grid nodes in the second inversion have an interval of 1º between 25º and 30º N and have a varying interval of 1.5º to 2º outside this region. We use the spherically symmetric global model ak135[2] as the initial regional velocity model. The regional model is embedded in a coarser (5° × 5°) global model that extends to 2889 km depth with an average depth interval of ~180 km (Supplementary Figure 3). We use the 3-D model LLNL_G3Dv3[3] for the initial global velocity model.

For the teletomoDD algorithm, large residuals are down-weighted by a bi-weight function[4-6]. A first-order smoothing constraint is applied to slowness perturbations to stabilize the tomographic system. The damped least squares system is solved by the LSQR algorithm[7]. The pseudo-bending ray tracing algorithm[8] that is extended to spherical coordinates[9] is used to calculate travel times. The damping and smoothing parameters are chosen via a trade-off

analysis of model perturbations and travel time residuals. For the coarser grid inversion, the root mean square (RMS) value of differential time residuals decreases from 1.089 s to 0.058 s after four simultaneous and two location-only iterations. In the first two simultaneous iterations, weights on absolute arrival time are set larger than differential arrival times to derive the large-scale structure. In the last two simultaneous ones, weights on differential arrival times are set much larger than the absolute arrival times to refine the near source structure. Supplementary Figure 4 shows cross sections of the inverted Vp model along six profiles in Figure 1.

In the second stage of the inversion, the initial velocity structure on the finer grid corresponds to the inverted Vp model using the coarser inversion grid. As for the coarser grid inversion, four simultaneous and two location-only iterations were performed and final RMS value of differential travel time residuals is 0.049 s, slightly lower than the coarser grid inversion. Figure 2 shows cross sections of the final Vp model using finer inversion grid along the 6 profiles in Figure 1. In comparison, the two models look very similar but the model from the 2nd stage of inversion with finer grid has larger anomaly amplitudes (Figure 2 and Supplementary Figure 4).

To evaluate the resolution of the Vp model around the subducting slab, we conducted checkerboard resolution tests[10]. Positive and negative 5% velocity anomalies are added to the 1D regional velocity model used in the inversion at alternating grid nodes to create the checkerboard velocity model. Synthetic absolute and differential travel times were calculated from the checkerboard velocity model using the same source-receiver geometry as the real data. Synthetic data were subsequently fed into the inversion system, with similar inversion parameters as the real data inversion. Supplementary Figures 5 and 6 show recovered checkerboard patterns for the coarser inversion grid at different depths and latitudes. Supplementary Figures 7 and 8 show recovered checkerboard patterns for the finer inversion grid. The checkerboard tests suggest that the inverted model is well-resolved horizontally and

vertically and that the high-velocity structure of slab in the Izu-Bonin area is well-defined below 100 km for the selected inversion grid. We also tried using finer inversion grid nodes in latitude with an interval of 1° from 8° N to 36° N. As shown by the checkerboard test results, the resolution becomes degraded compared to the inversion grid used for real data inversion (Supplementary Figure 9). For this reason, we think the currently selected inversion grid is a good compromise between resolution and robust retrieval of velocity anomalies. To quantitatively assess the resolvability of checkerboard models, we calculate the semblance value at each grid node between true and recovered checkerboard models using nearest nodes in three directions following the way of Guo et al.[11] which is modified from the method of Zelt[12]. We calculated the model resolvability for both coarser and finer grids. Supplementary Figures 10 and 11 show the distribution of resolvability values along the same 6 profiles as those in Figure 2 for coarser and finer grids, respectively. It can be seen from Supplementary Figures 10 and 11 that the model resolvability is high around and below the slab. However, in the shallower part above the slab and around the left and right edges of the model cross-sections, the model resolution is relatively poor with resolvability values < 0.7. These resolvability values are then used to determine the well-resolved model regions. For the coarser grid, the model region is treated as well resolved if the resolvability value at each grid node is larger than 0.8 (Supplementary Figure 4). For the model inverted using the finer inversion grid, we combine resolvability values obtained for coarser and finer grids to determine the well-resolved model region. This is because the model using finer inversion grid is inverted using coarser inversion grid model as the starting model so that any artifact from the coarser grid model would be brought onto the finer grid model. The model at finer grid node is treated as well resolved if the resolvability with the finer inversion grid is larger than 0.75 and the resolvability with the coarser inversion grid is larger than 0.8 (Figure 2).

In addition to the checkerboard resolution tests, we also conducted additional resolution tests by constructing various synthetic models with different characteristic features based on

the final inverted velocity model. Supplementary Figure 12 shows the synthetic model reflecting the main features of slab morphology resolved by the real data inversion, such as slab overturn to the south of 27° N (Figure 2 and Supplementary Figure 4). Here the synthetic model is constructed following high velocity anomalies of the inverted model. As a result, the "slab" in the synthetic model does not have smooth edges (Supplementary Figure 12). As for the checkerboard resolution test, we calculated absolute and differential travel times with the same distribution of sources and receivers as the real data. Then we applied the same inversion scheme as the real data for the synthetic data. It can be seen that the main features can be well recovered, but with a slightly lower amplitude (Supplementary Figure 13). This is expected because of the damping and smoothing regularizations used to stabilize the inversion system. This also indicates that the velocity anomalies in the inverted Vp model (Figure 2) are likely underestimated.

To further test the robustness of the low velocity anomaly around 400 km in depth with respect to the shallower and deeper parts of the slab appearing in the inverted model (Figure 2), we constructed a synthetic slab model similar to that in Supplementary Figure 12 but containing a low velocity zone around depth 400 km (Supplementary Figure 14). The inverted model shows that not only slab overturn can be well resolved but also the low velocity zone around 400 km can also be resolved (Supplementary Figure 15). Two other end-member slab models further show the robustness of main slab features resolved in the real data inversion (Supplementary Figures 16-19). The model in Supplementary Figure 17 shows that slab overturn cannot be retrieved as an inversion artifact. The model in Supplementary Figure 19 shows that the inversion procedure can also recover the case where the slab overturns throughout the area. In the slab model shown in Supplementary Figure 16, we include the (relatively low) velocity anomaly in the slab around depth 400 km. This feature is also well resolved (Supplementary Figure 17). These synthetic tests show that the velocity

anomalies that we discuss in the main text are robust and can be resolved by the real data (Figure 2).

The last resolution test that we conducted is the restoration test[13], which is a traditional and widely used method for model resolution estimation. It can directly evaluate the reliability of the shape and amplitude of velocity anomalies from the real data inversion as well as some features seen from earthquake relocations. Firstly, we set earthquake relocations and velocity models obtained from the real data inversion as "true" earthquake locations and "true" velocity models (Figure 2). The synthetic travel times are then calculated based on these "true" earthquake locations and velocity models. We then add Gaussian distributed noise of mean of zero and standard deviation of 1 second to the synthetic travel times. The same inversion strategy is applied to these synthetic travel times as the real data. It can be seen that the recovered model (Supplementary Figure 20) is very similar to the inverted model shown in Figure 2, with the RMS difference of 0.0409km/s. For the 2015 Bonin earthquake, the restored location differs by 489 m in longitude, 1861 m in latitude, and 1382 m in depth from the "true" location, indicating that the 2015 Bonin earthquake is well located.

To further check the robustness of the main slab features, we conducted a bootstrap analysis by randomly discarding 10% of data for inversion. We repeated this process for 50 times and calculate the standard deviations for the percentage change of the inverted model with respect to the ak135 model[1] for each grid node. It is found that at most grid nodes, the standard deviation is smaller than 0.04%, suggesting model uncertainties are small (Supplementary Figure 21). In comparison, below 660 km where earthquakes are scarce, model uncertainties are slightly larger and around 0.05-0.06%.

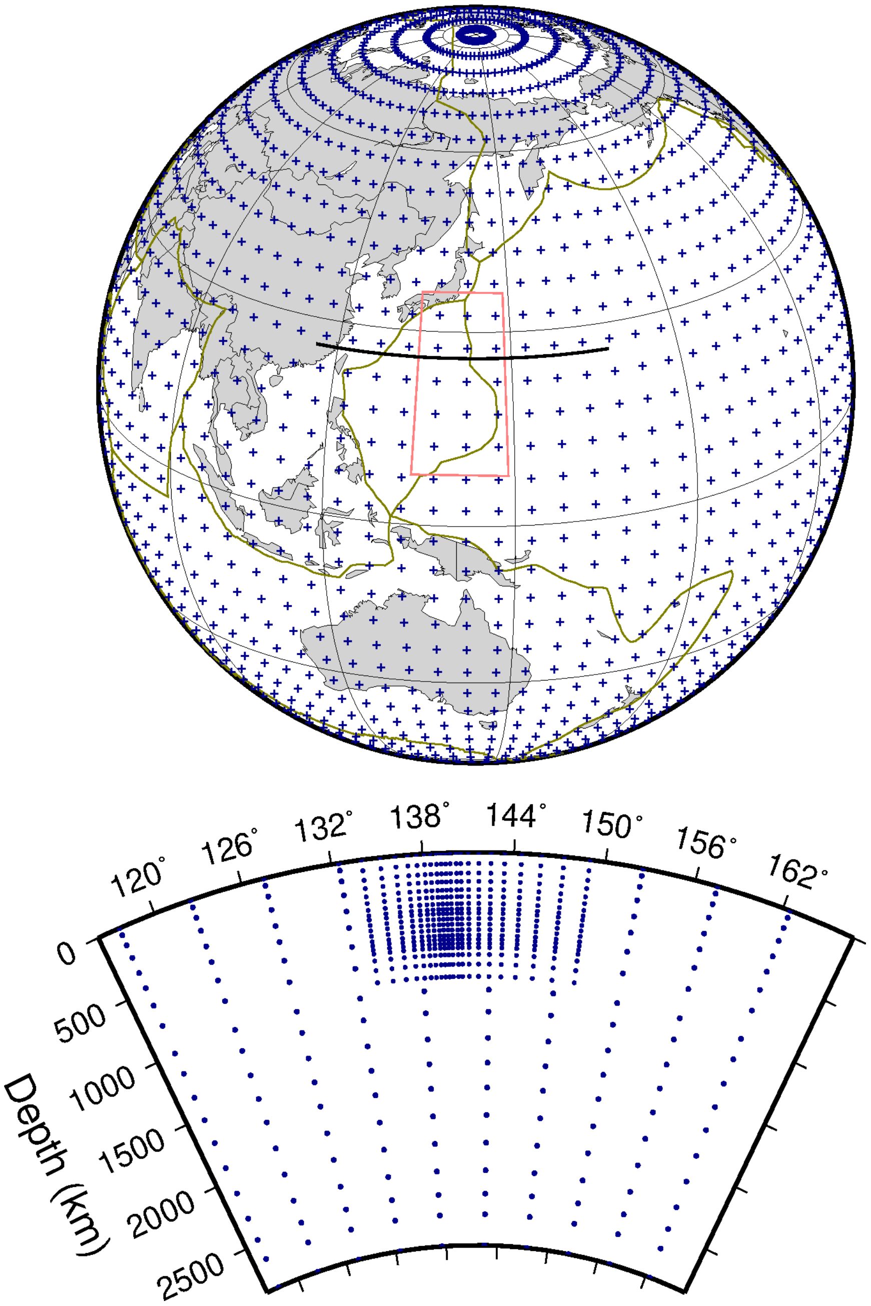

Supplementary Figure 3: Nested regional-global inversion grids used for the Izu-Bonin tomography. The upper panel shows the inversion grid (blue pluses) for the global model, and the red rectangle marks our study region. The lower panel shows the inversion grid for the regional model enclosing the Izu-Bonin subduction zone. The lateral inversion grid node spacing for the global model is 5º, while it varies from ~1.2º near the edge of the study region to 0.3º near the 2015 Bonin earthquake in longitude

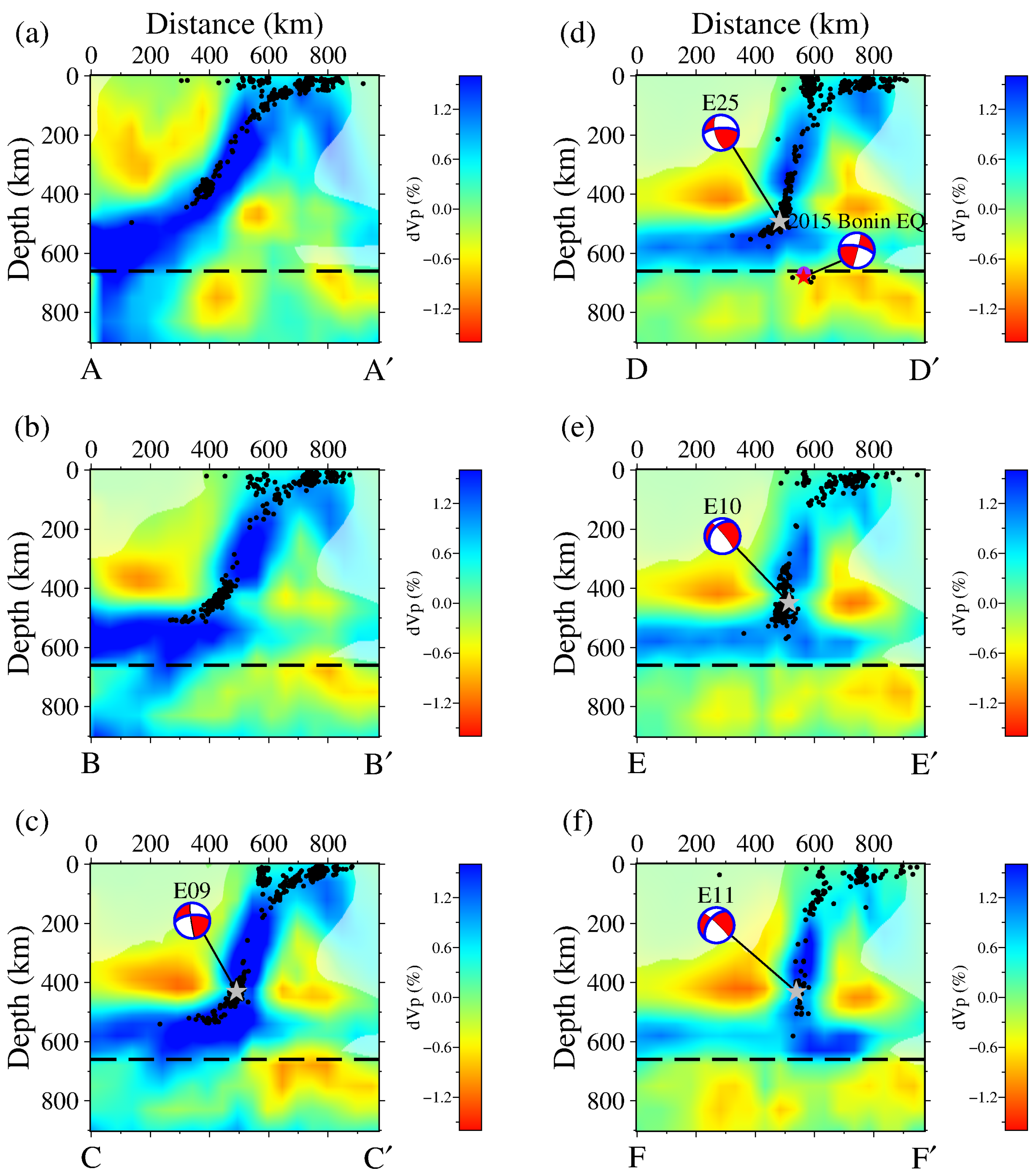

Supplementary Figure 4: The same as Figure 2 but the inverted model is obtained using the coarser inversion grid. The model region with resolvability less than 0.8 is masked.

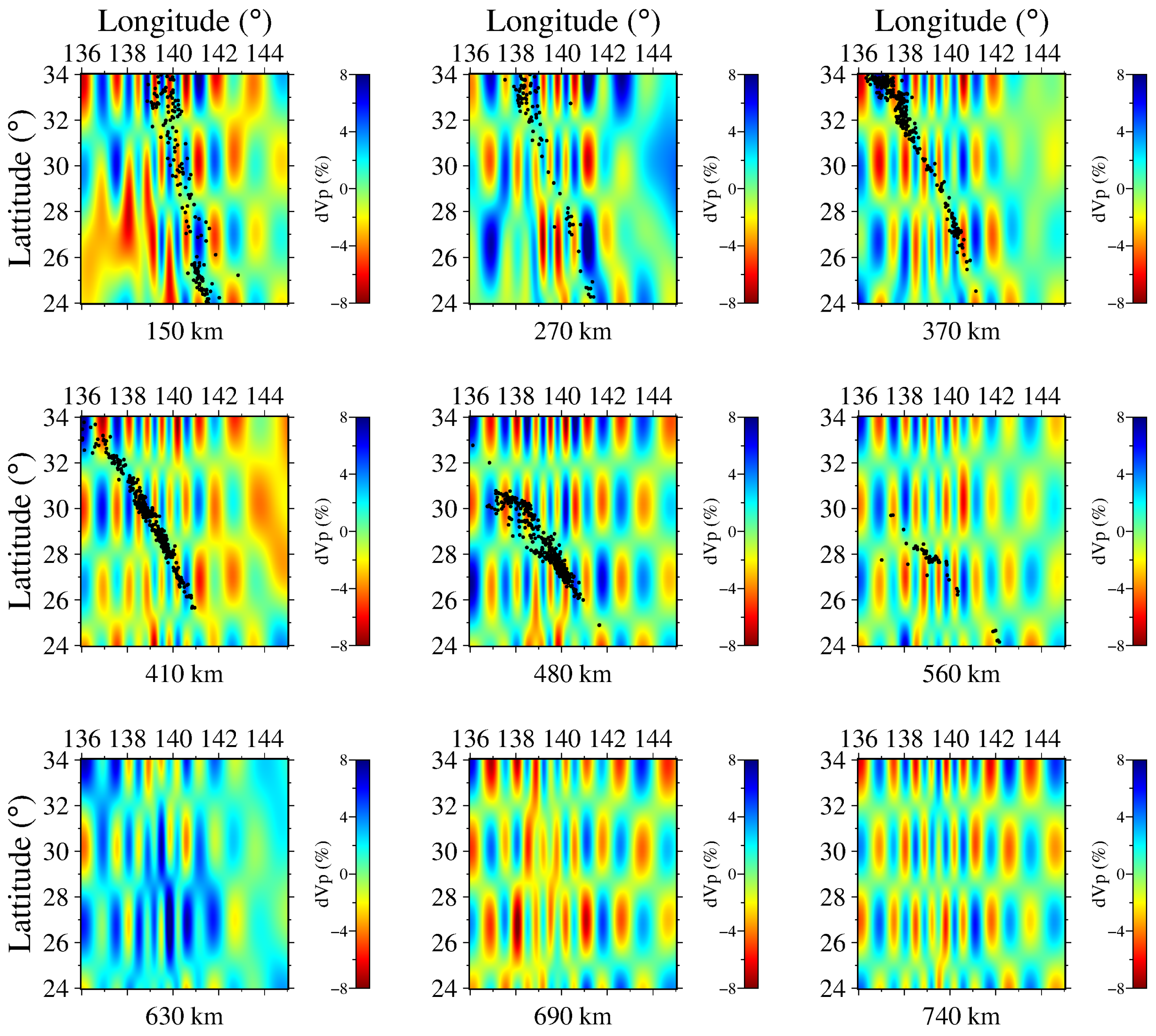

Supplementary Figure 5: Horizontal slices of recovered checkerboard patterns at different depths for the coarser inversion grid.

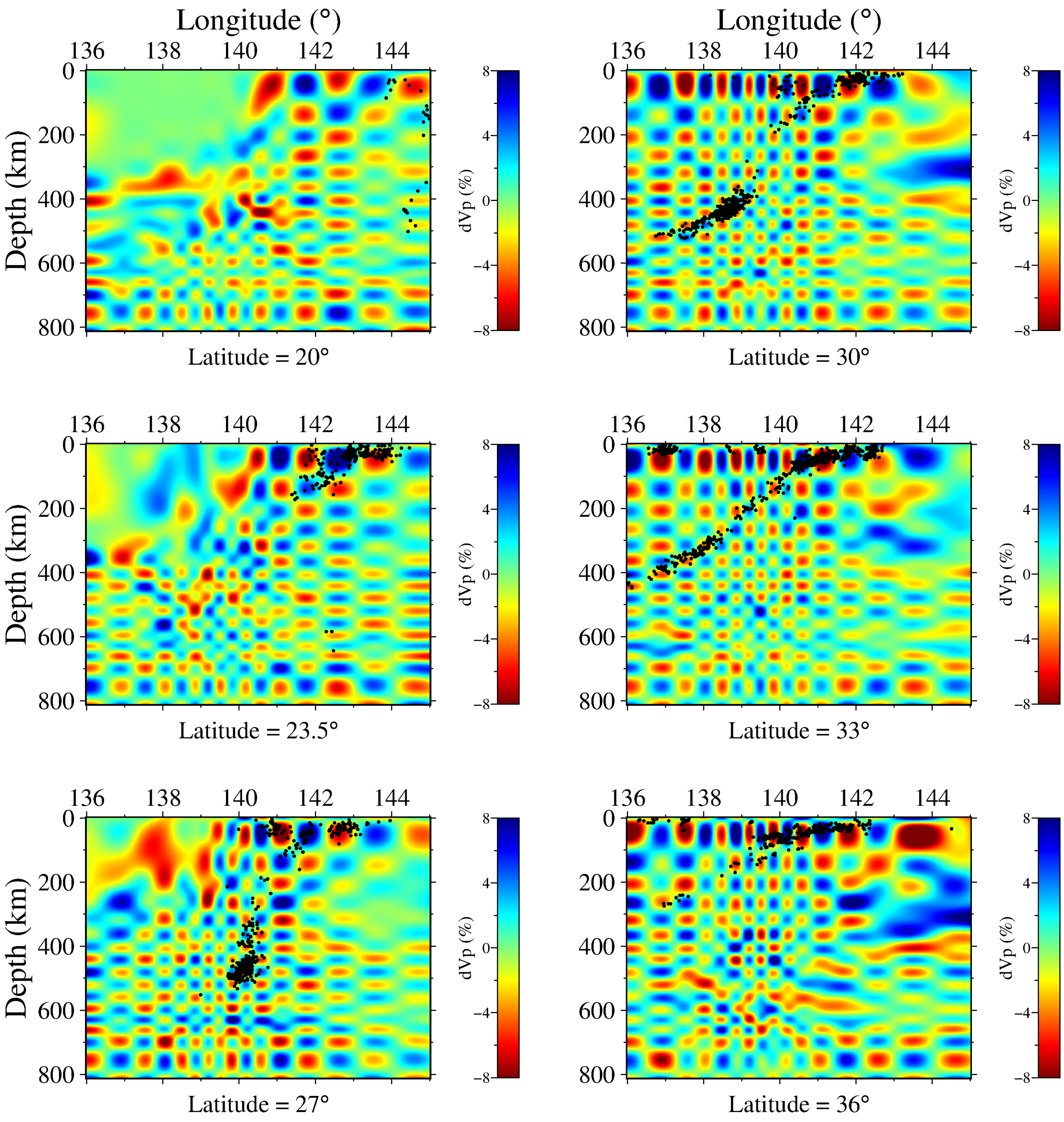

Supplementary Figure 6: Recovered checkerboard patterns at selected latitudes in the case of coarser inversion grid.

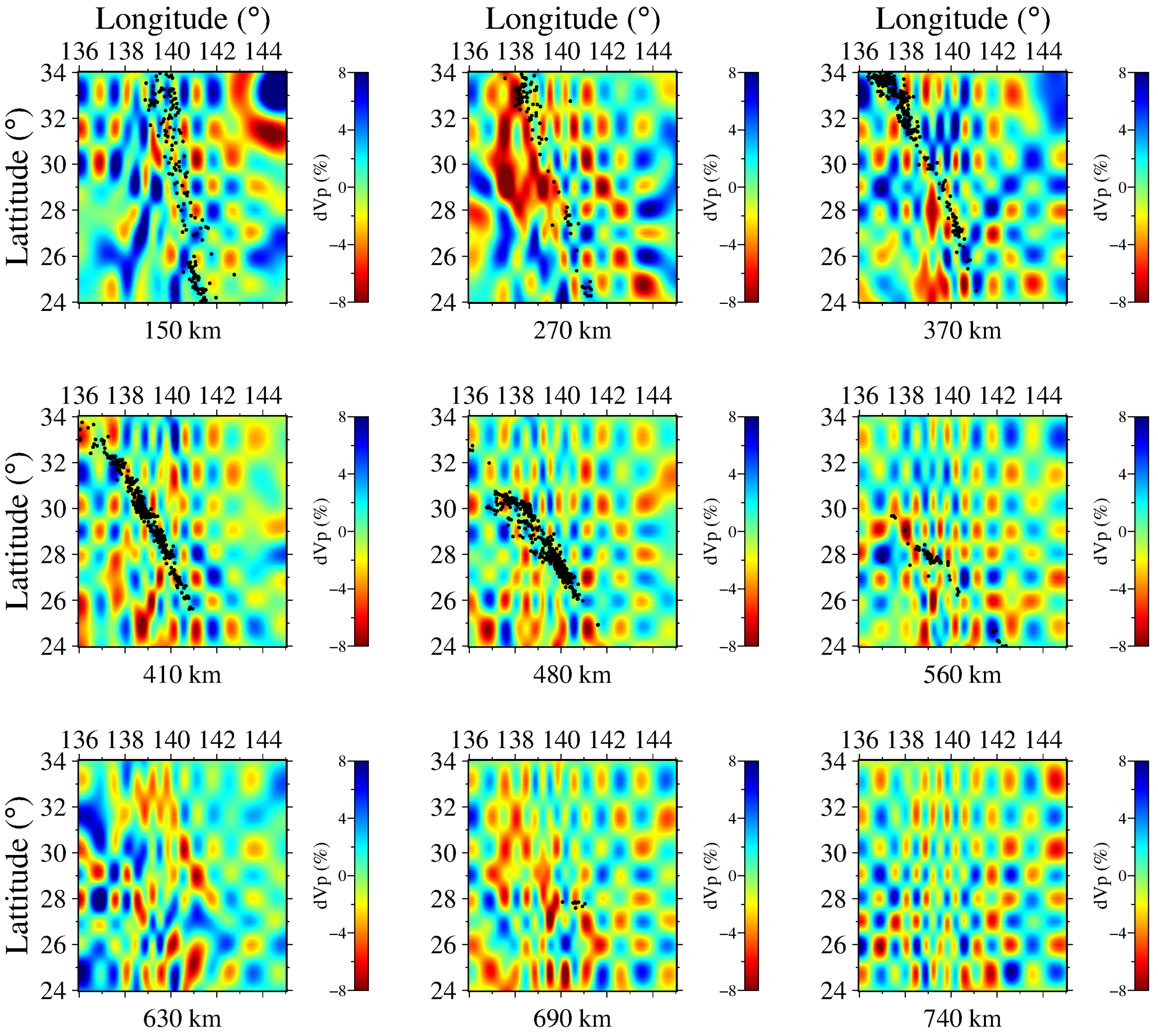

Supplementary Figure 7: Horizontal slices of recovered checkerboard patterns at different depths for the finer inversion grid.

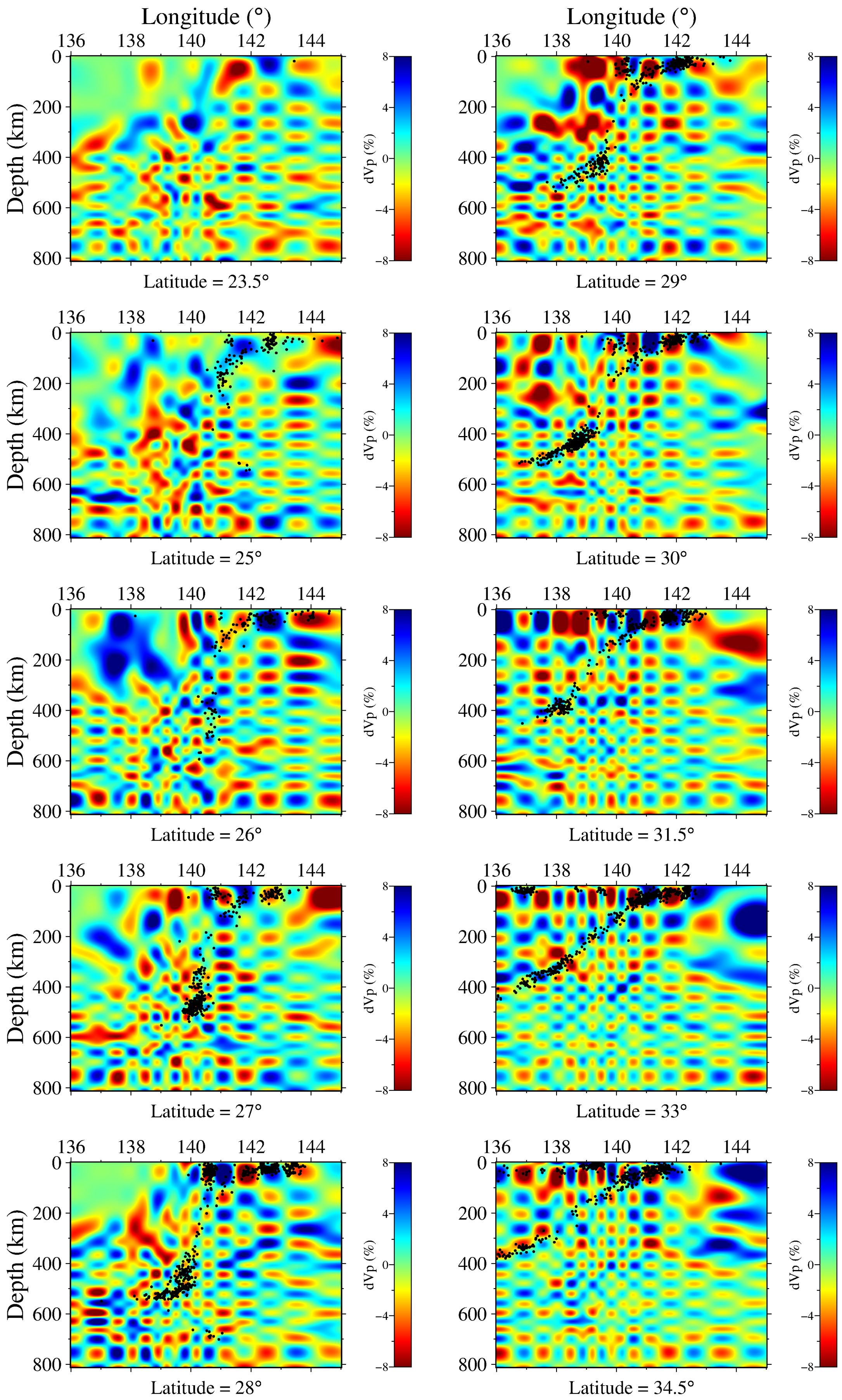

Supplementary Figure 8: Recovered checkerboard patterns at selected latitudes in the case of finer inversion grid.

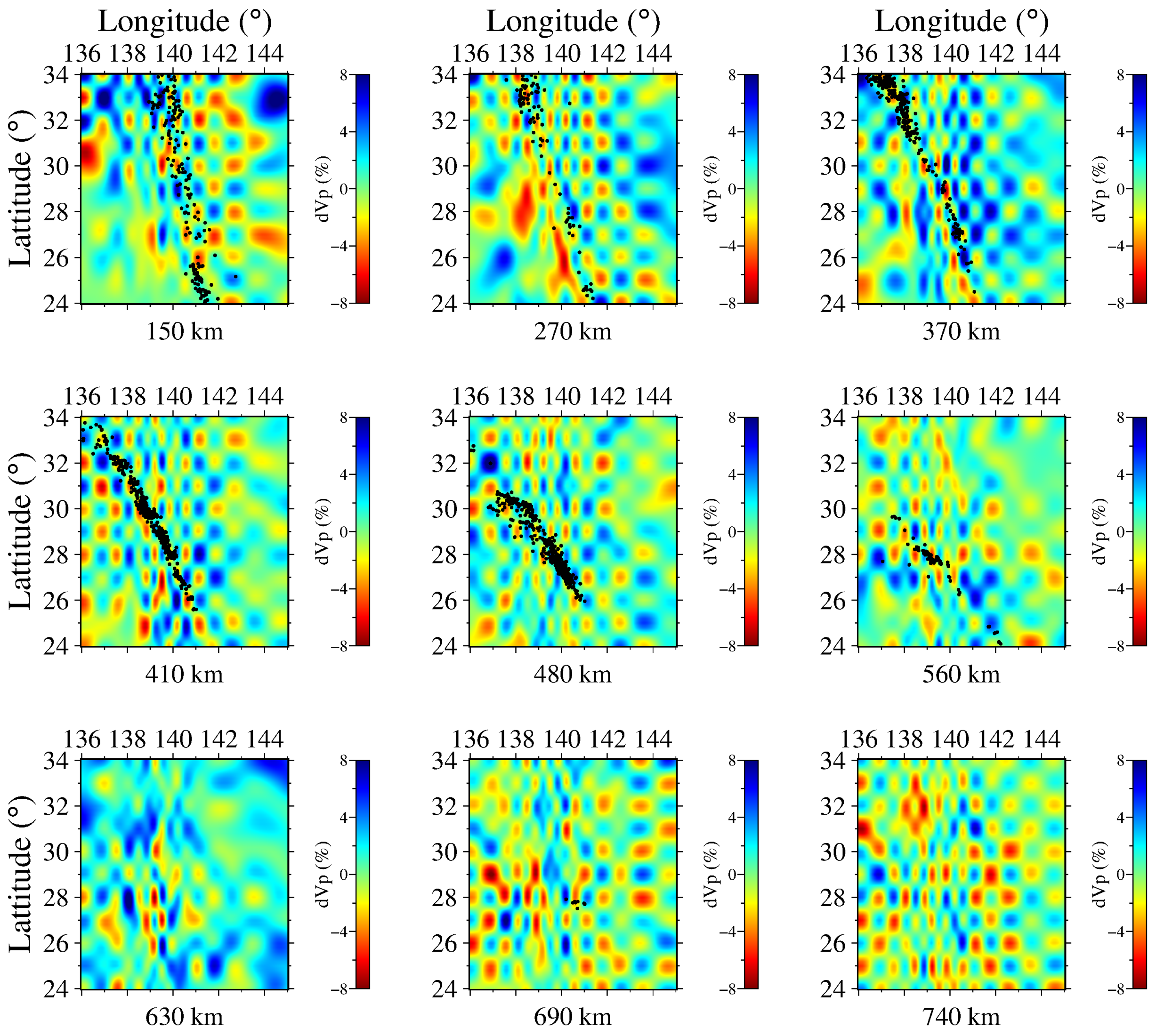

Supplementary Figure 9: Horizontal slices of recovered checkerboard patterns at different depths in the case the inversion grid interval is 1° in latitude for the regional model.

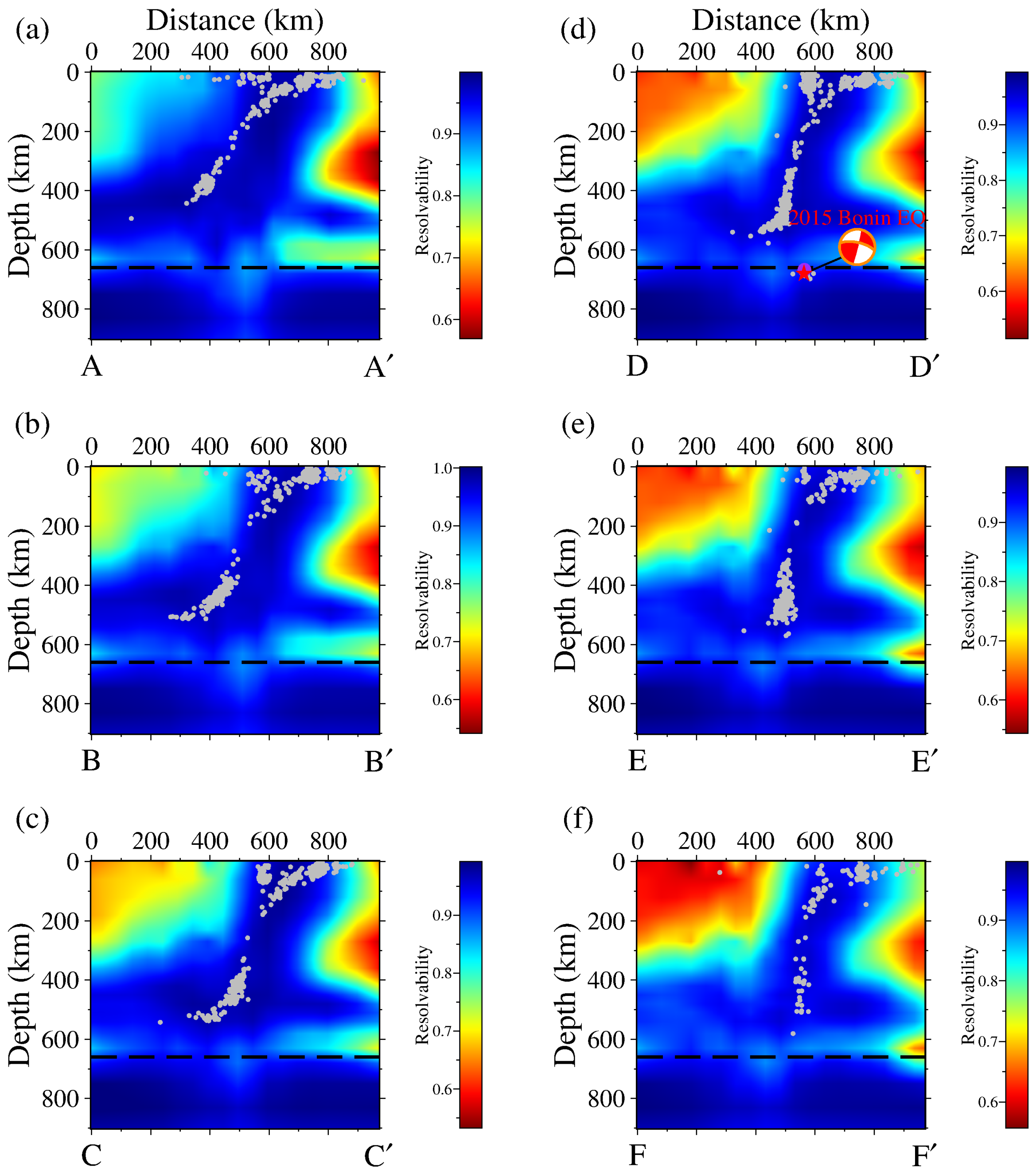

Supplementary Figure 10: Distribution of resolvability (or semblance) values in the case of coarser inversion grid along 6 profiles shown in Figure 1.

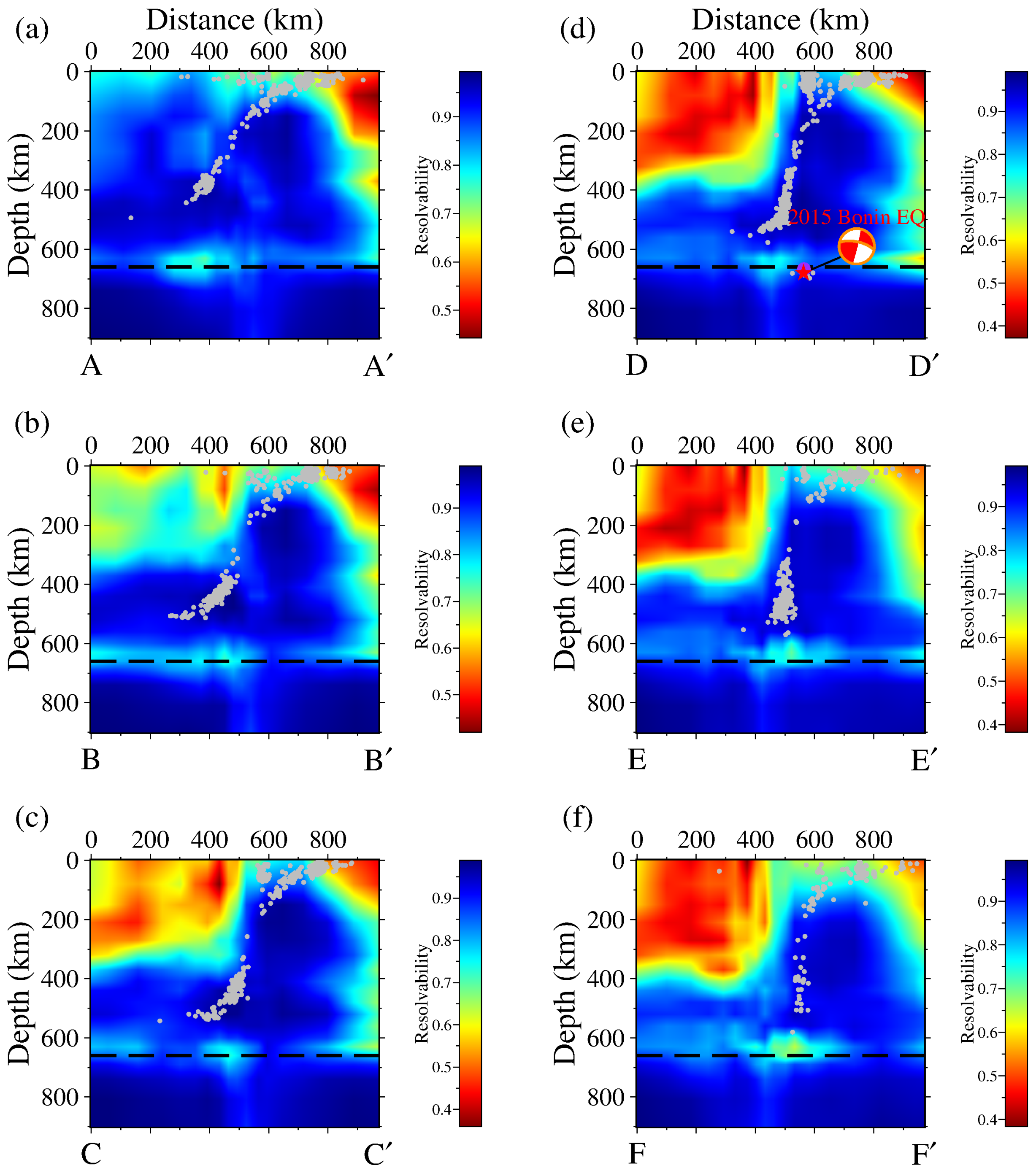

Supplementary Figure 11: Distribution of resolvability (or semblance) values in the case of finer inversion grid along 6 profiles shown in Figure 1.

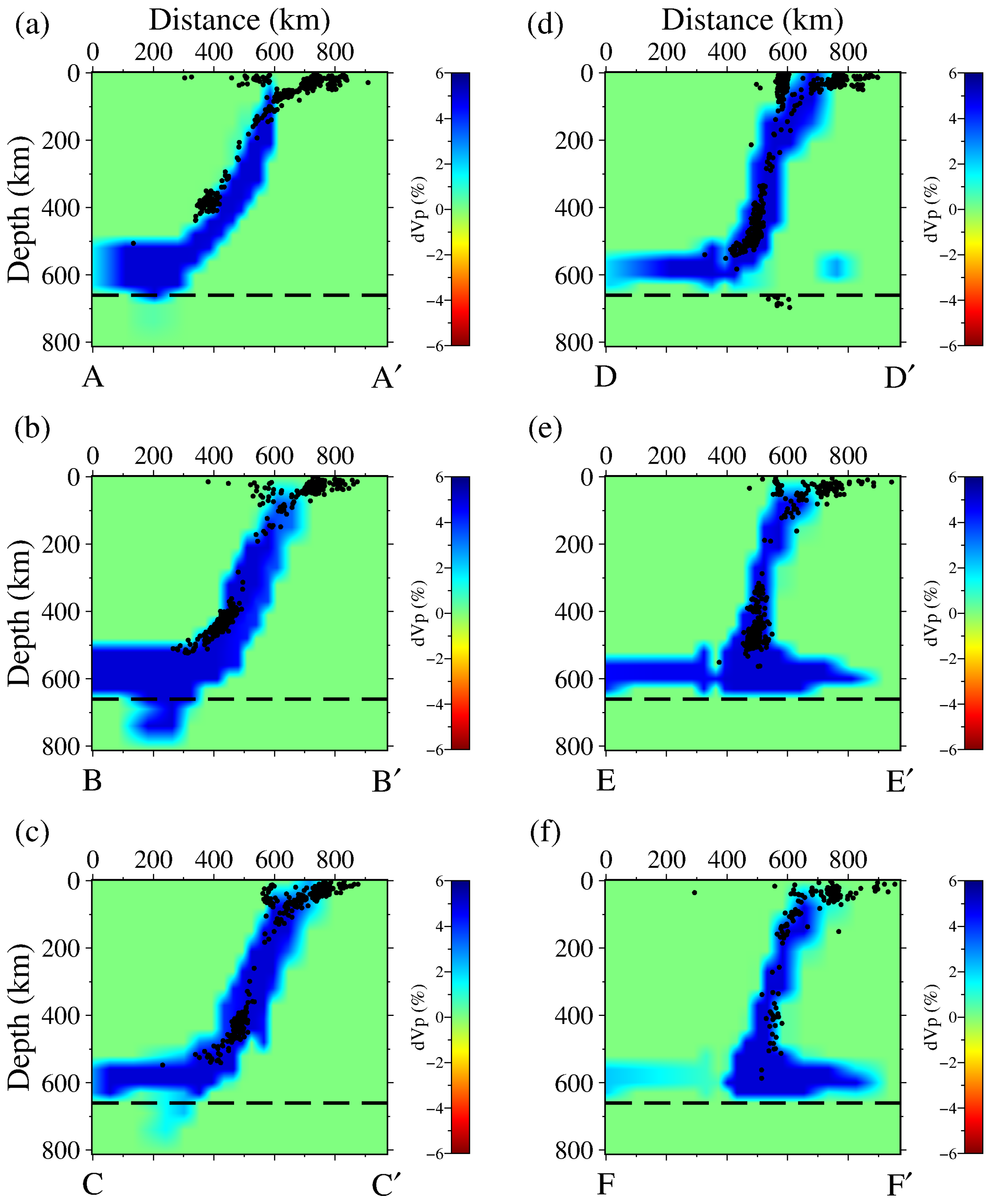

Supplementary Figure 12: Synthetic model with the similar slab morphology as the inverted model from real data shown in Figure 2. The profiles are shown in Figure 1.

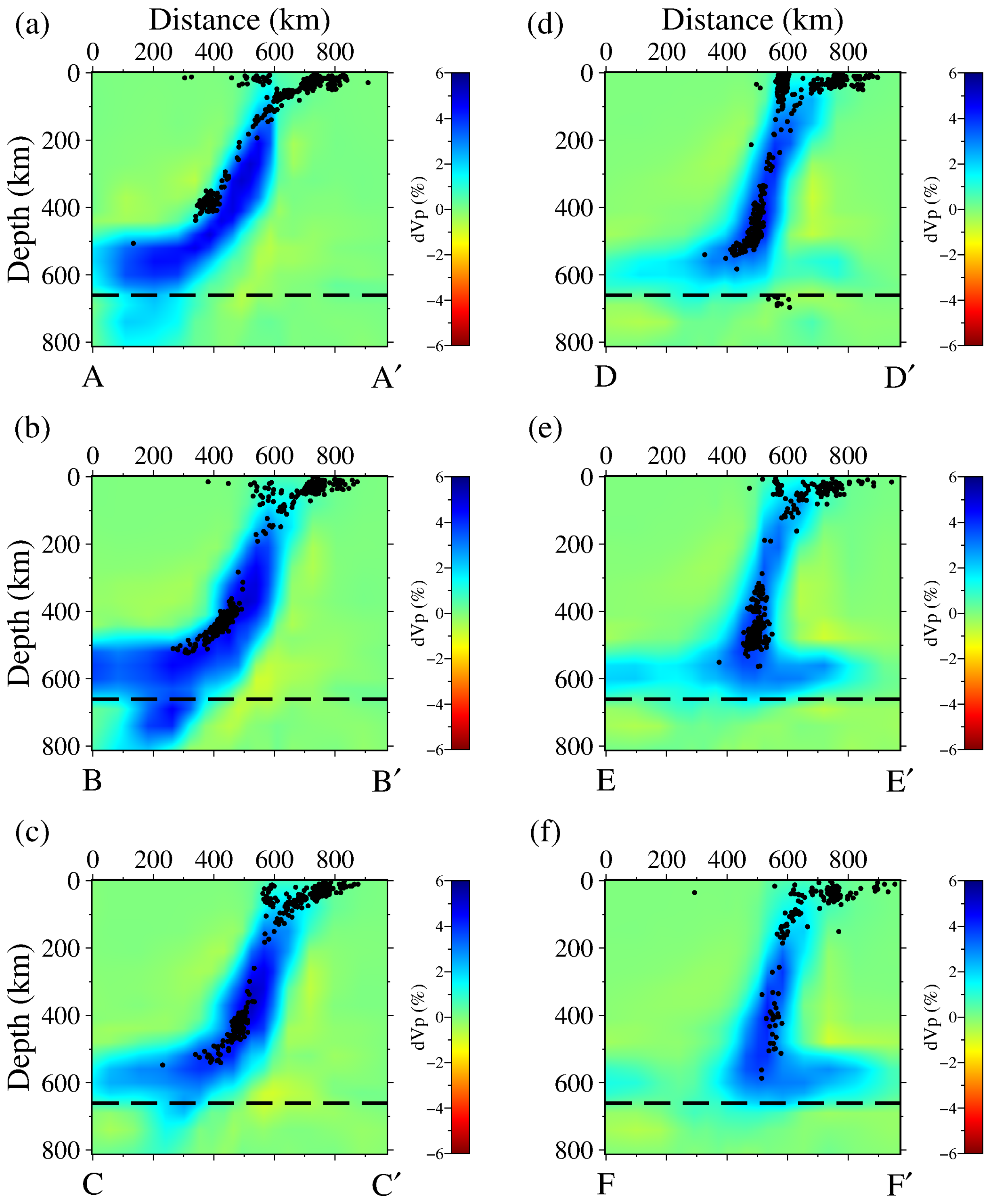

Supplementary Figure 13: Recovered model using the same inversion procedure as the real data for the synthetic model shown in Supplementary Figure 12. The profiles are shown in Figure 1.

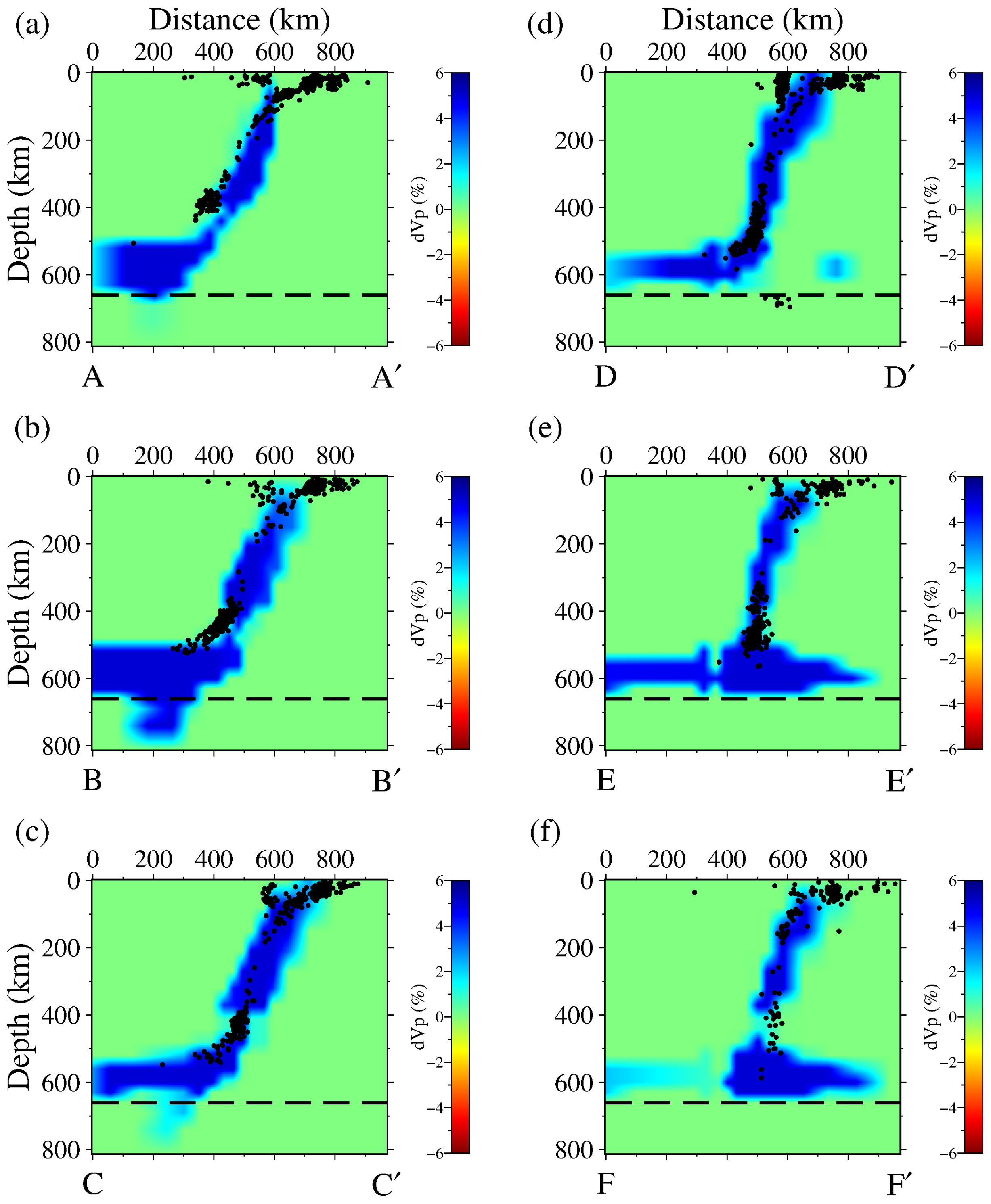

Supplementary Figure 14: The same as Supplementary Figure 12 but the slab contains a weak zone around 400 km. The profiles are shown in Figure 1.

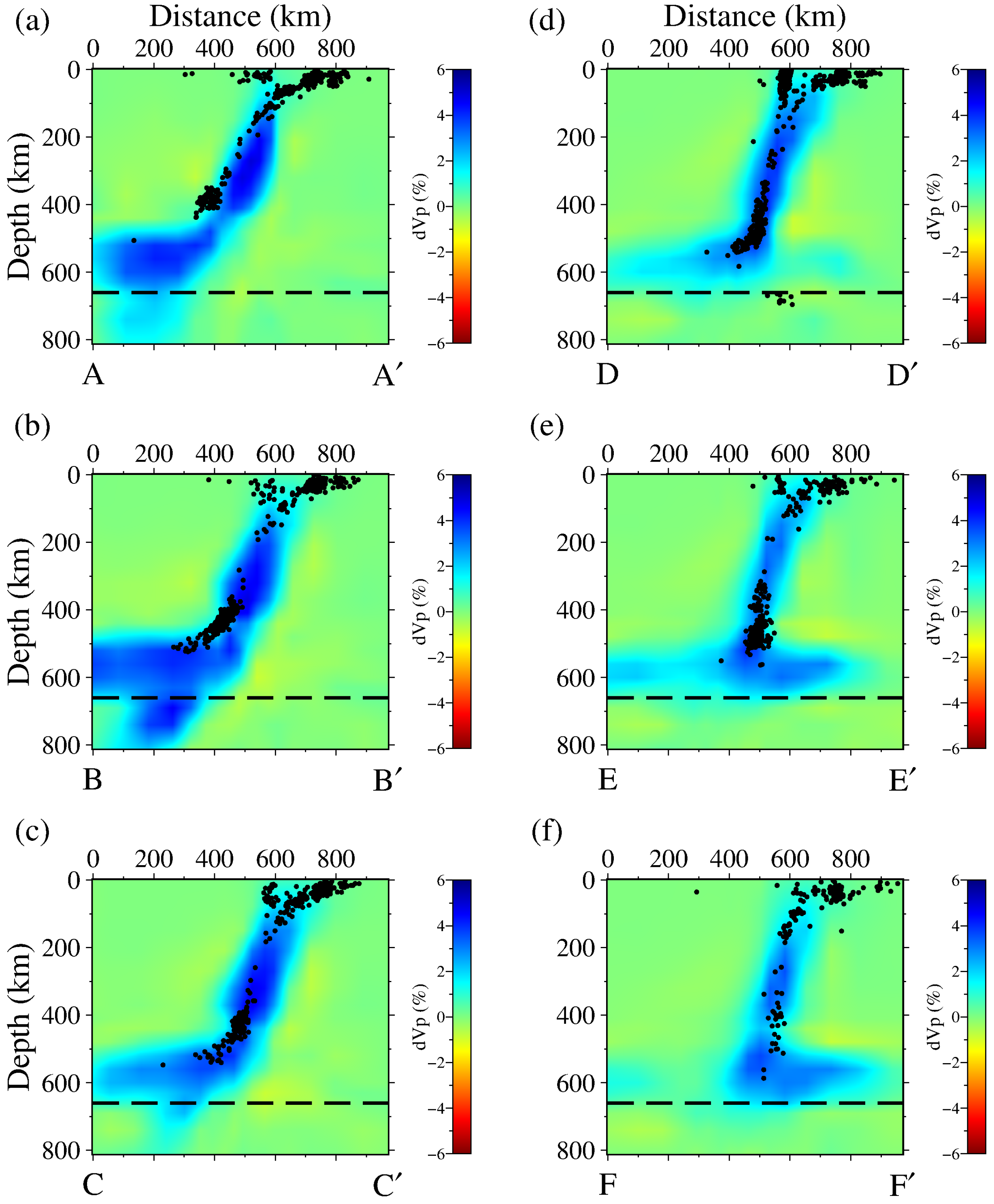

Supplementary Figure 15: Recovered model using the same inversion procedure as the real data for the synthetic model shown in Supplementary Figure 13. The profiles are shown in Figure 1.

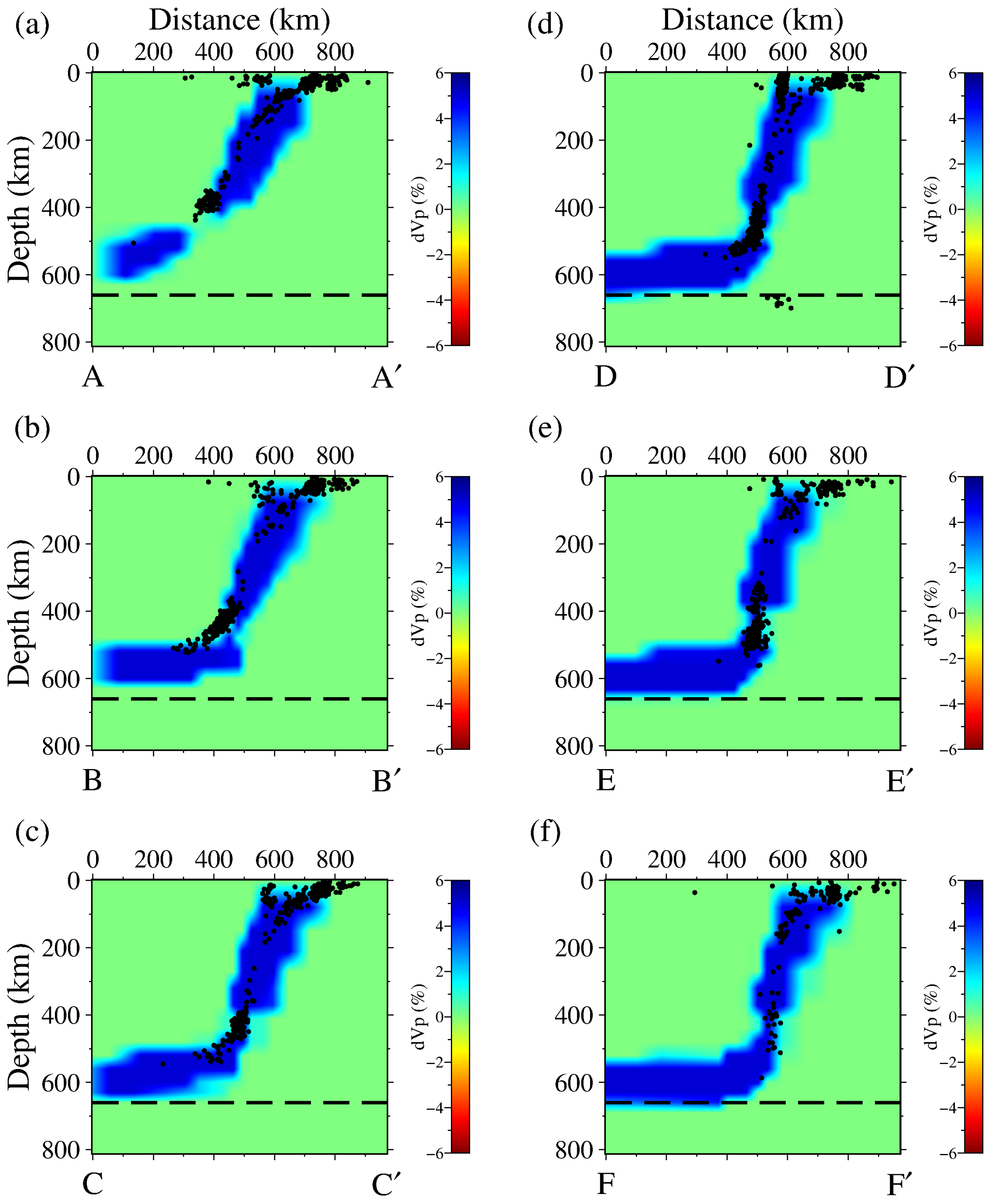

Supplementary Figure 16: Synthetic model with slab dipping towards to west and having a weak zone around 400 km. The profiles are shown in Figure 1.

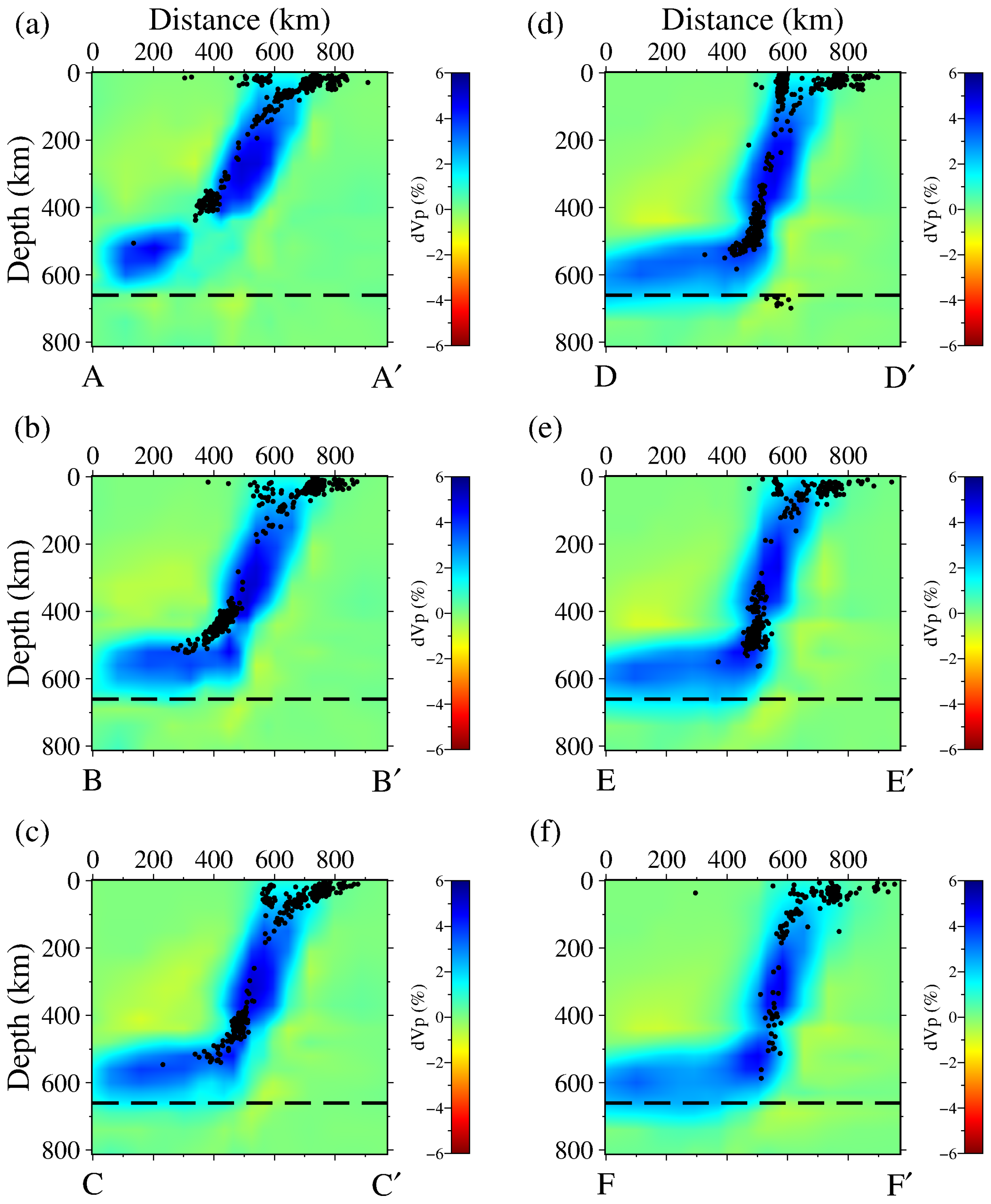

Supplementary Figure 17: Recovered model using the same inversion procedure as the real data for the synthetic model shown in Supplementary Figure 16. The profiles are shown in Figure 1.

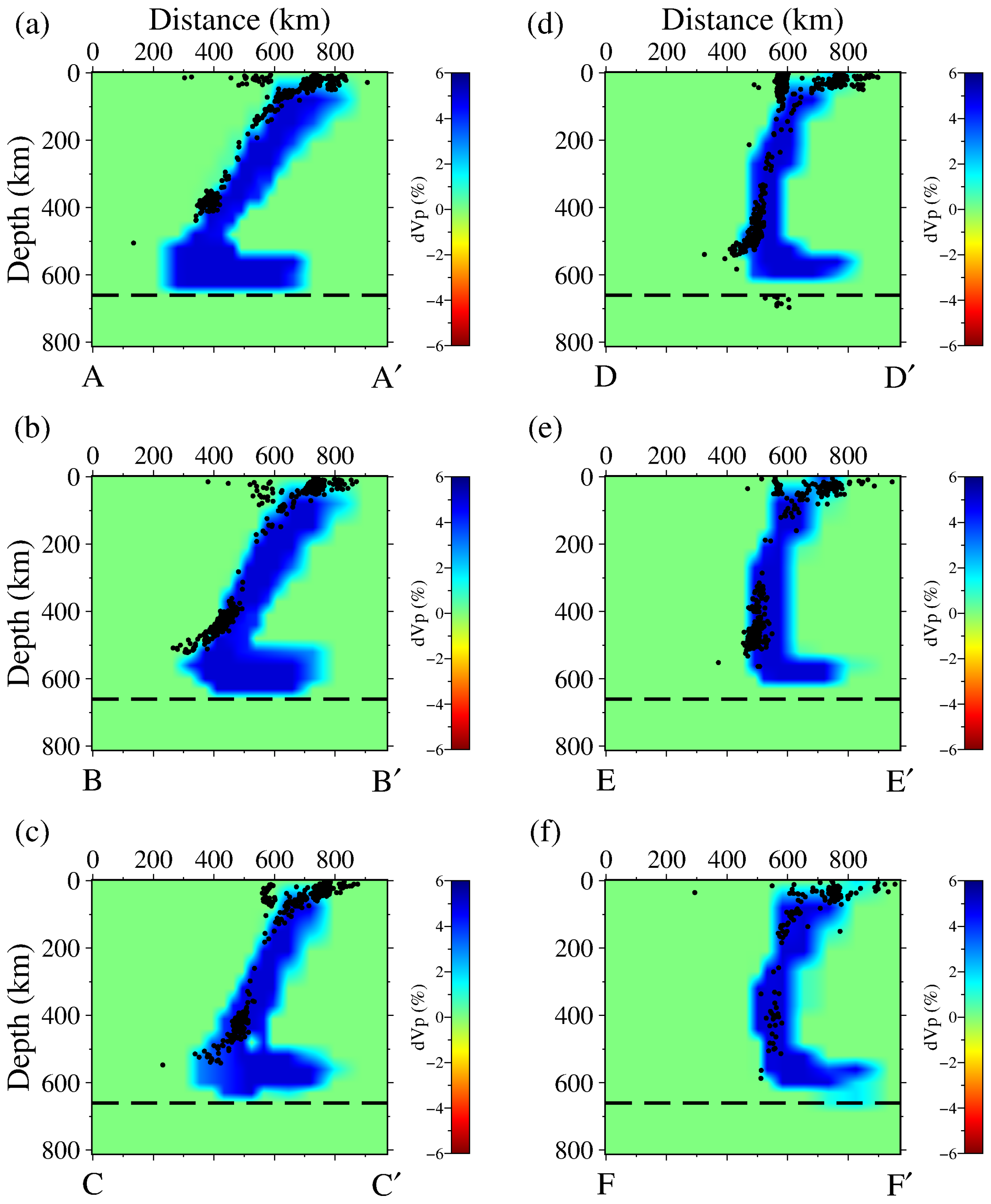

Supplementary Figure 18: Synthetic model with slab overturned. The profiles are shown in Figure 1.

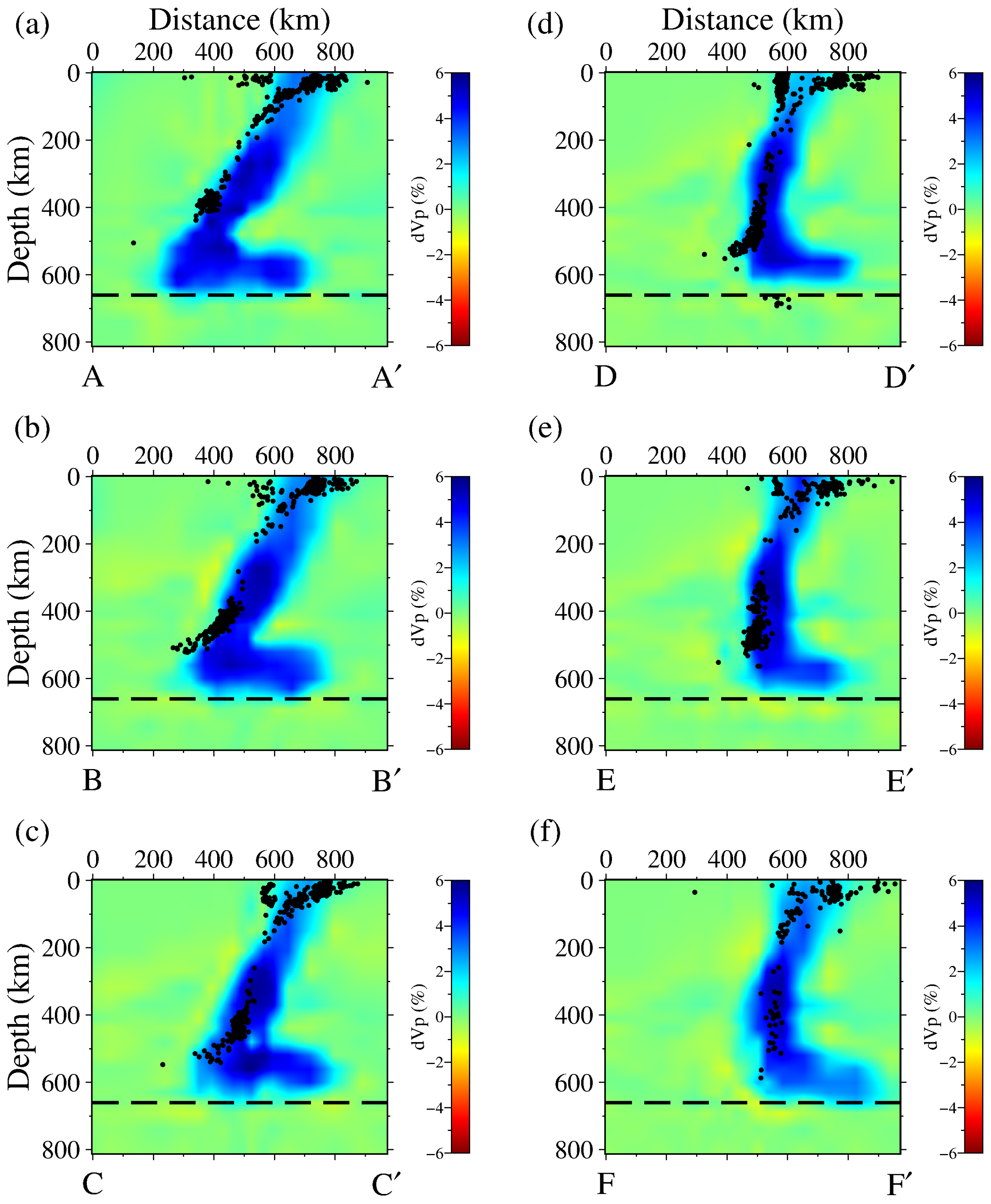

Supplementary Figure 19: Recovered model using the same inversion procedure as the real data for the synthetic model shown in Supplementary Figure 18. The profiles are shown in Figure 1.

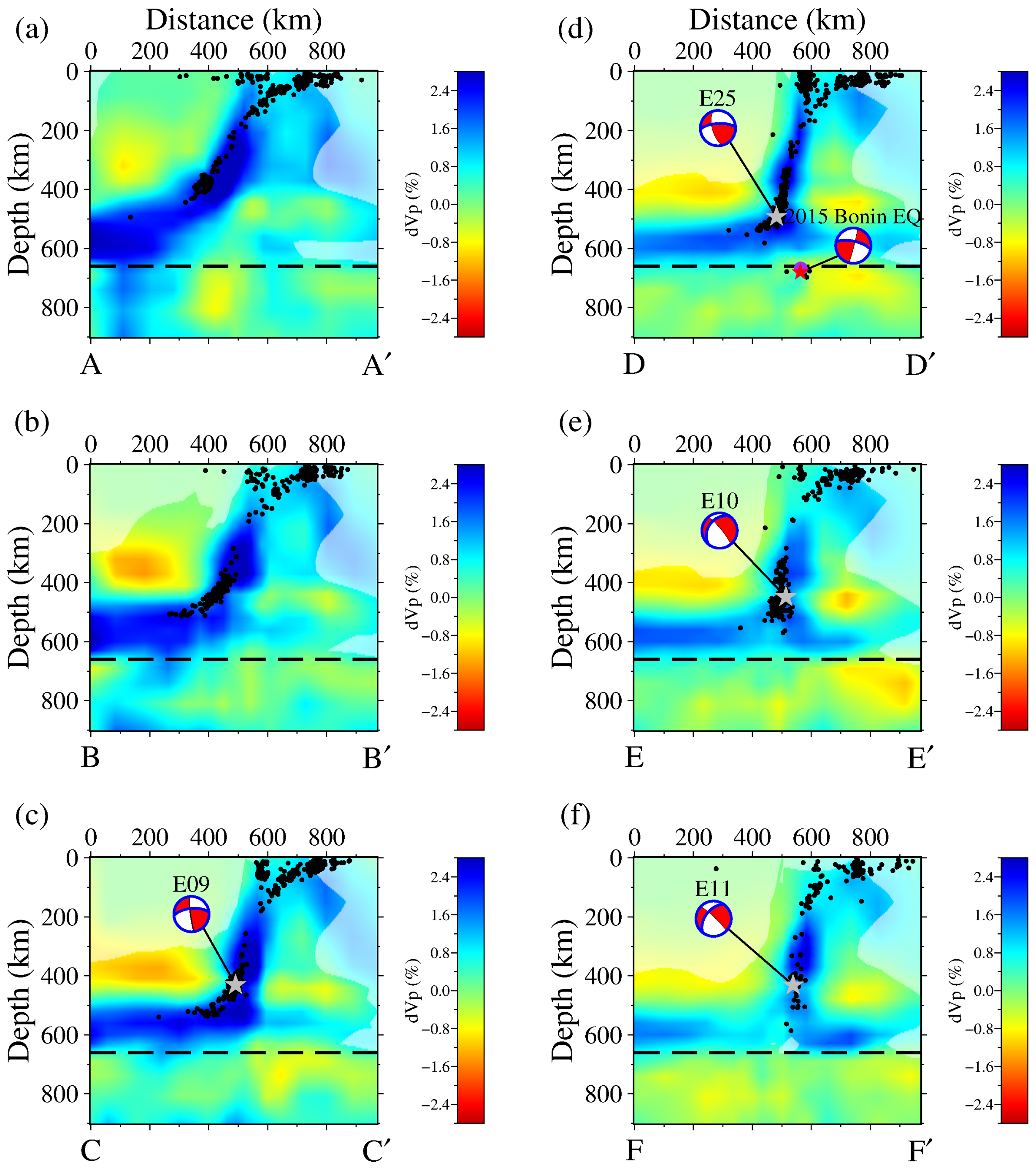

Supplementary Figure 20: Cross sections of the inverted Vp model from the restoration resolution test for the model shown in Figure 2. The profiles are shown in Figure 1.

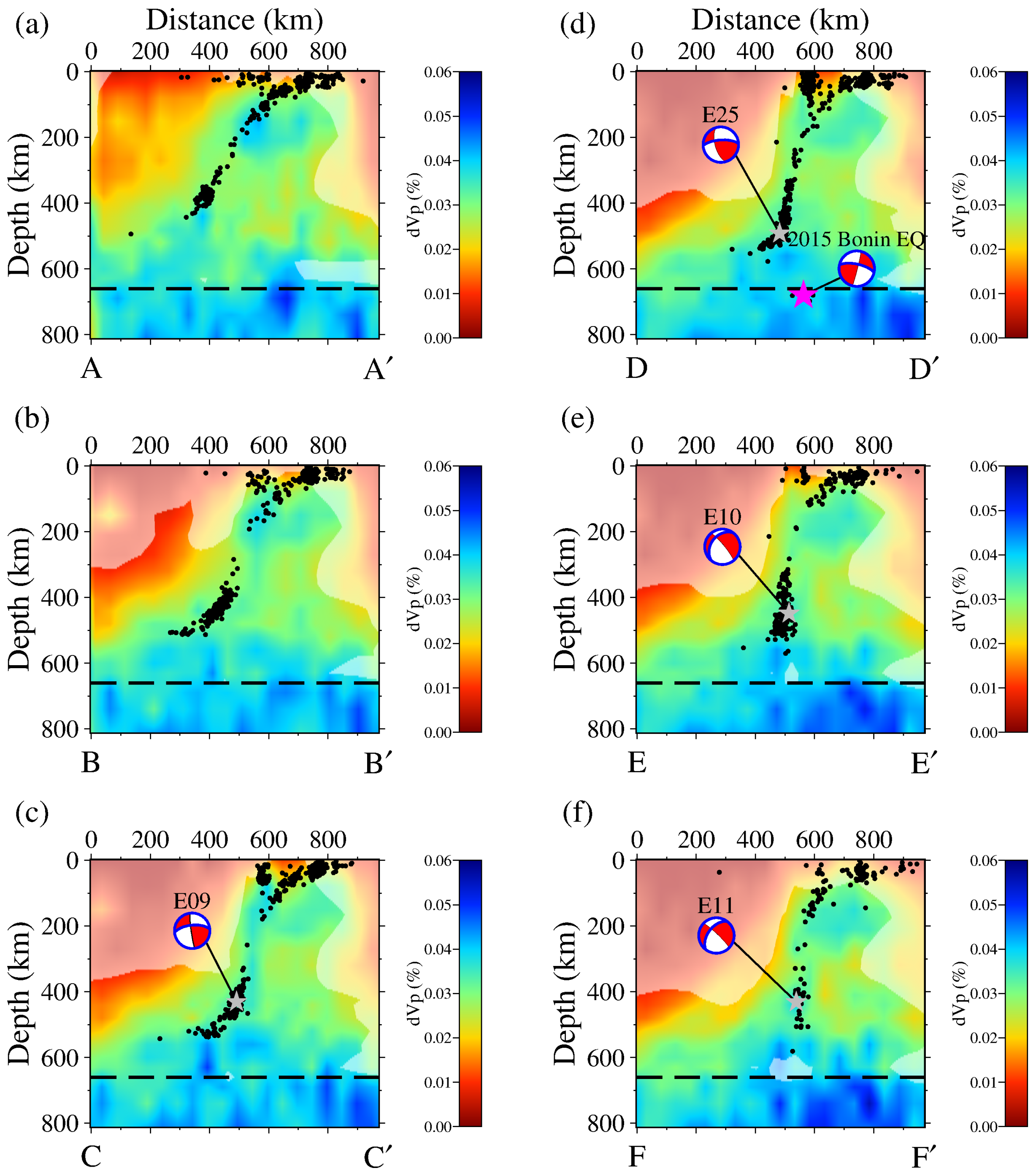

Supplementary Figure 21: Standard deviations for the velocity perturbation with respect to the AK135 model for the inverted model shown in Figure 2, obtained from the bootstrap analysis. The profiles are shown in Figure 1.

Supplementary Note 2 Estimating temperature increase and viscosity within a slab shear zone

To estimate temperature increase and viscosity within a 25 km thick shear zone at 13 GPa (~420 km) observed from the inverted model (Figure 2), we give the flow law parameters in Supplementary Table 1 that are used by the method of modeling viscous dissipation. In the absence of quantitative estimates for wadsleyite and ringwoodite, we use dry olivine flow laws for diffusion and dislocation creep[14], and the Peierls' creep parameters[15]. Activation volumes are expected to be on the order of 10 cm^3/mol at the surface, decreasing with increasing pressure; the constant value of 6 cm^3/mol chosen here is the average of the bounds[14], and is probably a reasonable average for the upper mantle. Supplementary Figure 22 shows the amount of heating in a 25 km thick shear zone under a variety of strain rates.

Supplementary Note 3 The effect of phase transitions and metastability on seismic velocities, and relevance to the proposed slab shear zone

Solid-solid phase transitions will influence the P-wave velocity difference between the slab and surrounding mantle. The most prominent reactions are expected to increase velocities within the slab relative to the mantle. For example, the equilibrium transformations from olivine to wadsleyite and wadsleyite to ringwoodite are associated with positive velocity jumps, and both reactions have positive Clapeyron slopes (e.g. Stixrude and Lithgow-Bertelloni[16]). Therefore, they are expected to result in an increase in slab velocities at a given pressure relative to the ambient mantle. The presence of metastable olivine within a cold slab could lower velocities relative to surrounding mantle at depths greater than the "410 km" transition[17] (Supplementary Figure 23).

With respect to the proposed shear zone in the Izu-Bonin slab, there is little change in predicted slab temperatures along the slab based on plate age and subduction velocity

alone[19]. Therefore, major changes in velocity structure along the slab are unexpected. In particular, the ability to metastably preserve olivine is unlikely to be very different in the south relative to the north. We therefore prefer to explain the localised reduction of velocities about the tight fold in the slab as a consequence of deformation, rather than differences in phase assemblage resulting from different thermal conditions.

Parameter	Diffusion creep	Dislocation creep	Peierls' creep
A_i ($\mathrm{m}^{p_i}\mathrm{Pa}^{-n_i}/\mathrm{s}$)	2.25×10^{-15}	6.51×10^{-16}	3.6×10^{-19}
n_i	1	3.5	2
p_i	3	0	0
E_{iact} (J)	3.75×10^{5}	5.3×10^{5}	3.2×10^{5}
V_{iact} (m^3)	6×10^{-6}	6×10^{-6}	6×10^{-6}
σ_i (Pa)	-	-	5.9×10^{9}
q_i	-	-	0.5
s_i	0	0	1

Supplementary Table 1: Flow law parameters for the shear heating model.

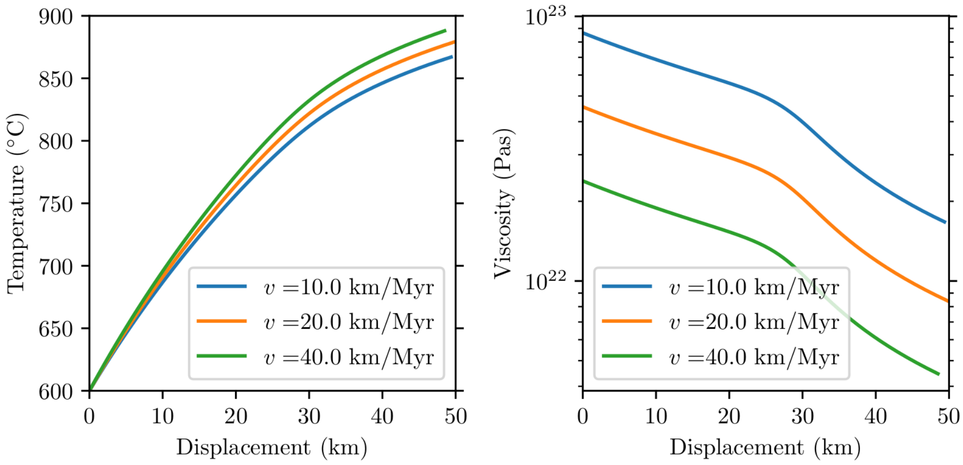

Supplementary Figure 22: Estimates of temperature increase and viscosity within a 25 km thick shear zone at 13 GPa (~400 km depth). The flow laws used in the calculations correspond to experimental values for dry olivine[14,15].

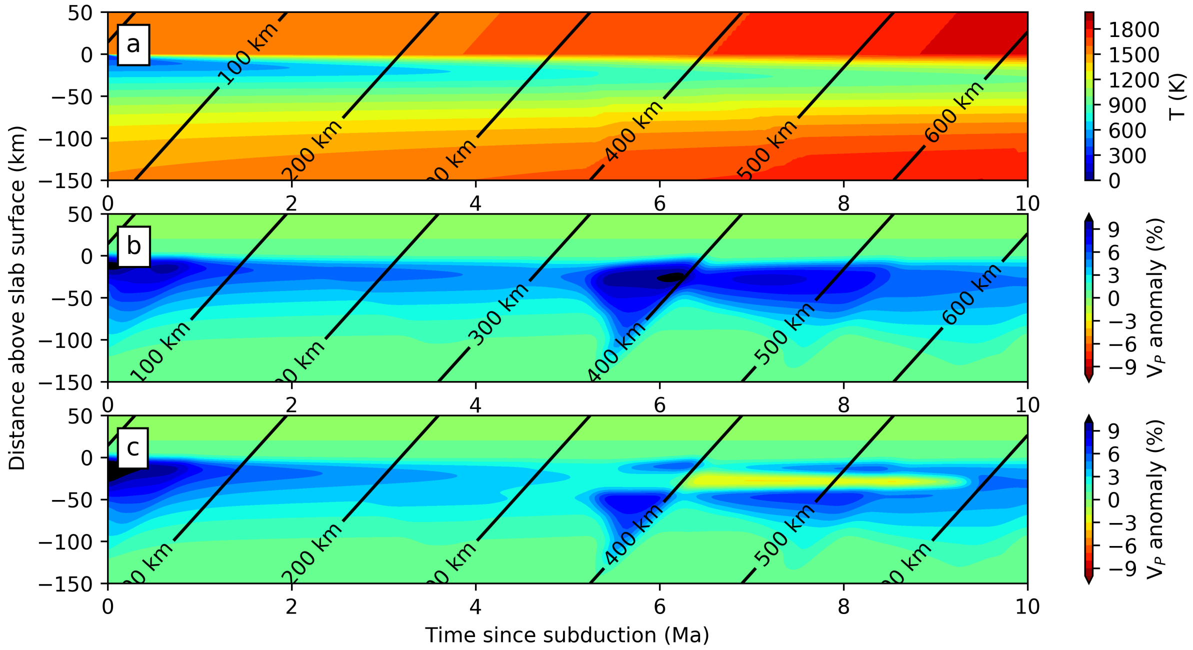

Supplementary Figure 23: The effect of slab temperature and metastability on seismic anomalies relative to ambient mantle. a) Simplified slab temperature structure (in Kelvin) calculated according to the model in Frohlich[18]. The age at the trench is 130 Myr, the velocity is 7 cm/yr, the diffusivity 5e-7 m^2/s, and the slab dip is 60 degrees. Black contours correspond to depths in km. Temperatures are obtained from analytically-calculated potential temperatures by adding a component due to adiabatic heating, using a PerpleX lookup table for pyrolite (the choice of depleted or undepleted slab material has little influence on the results). b) Equilibrium P-wavespeeds for a pyrolitic slab in km/s. c) P-wavespeeds for a model where olivine remains metastable below 973 K. For ease of calculations, a simplified harzburgite composition is used wherever the slab temperature is <973 K. This is obviously non-physical (it implies that slab material "magically" transforms from harzburgite to pyrolite at 973 K), but simplifying the bulk composition simplifies the definition of metastability (allowing Fe-Mg exchange in olivine and pyroxene, but inhibiting transformation to wadsleyite, ringwoodite and majoritic garnet) while having only a small effect on slab velocities (see the ~1% difference in velocities between (b) and (c) at <300 km). Metastable olivine is visible as a wedge of anomalously low velocities between 400-600 km depth. For both (b) and (c), velocities have been smoothed with a Gaussian filter with sigma= 5 km.

Supplementary References

1. Engdahl, E. R., van der Hilst, R., & Buland, R. Global teleseismic earthquake relocation with improved travel times and procedures for depth determination. *Bull. Seism. Soc. Am.* **88(3)**, 722-743 (1998).
2. Kennett, B. L. N., Engdahl, E. R., & Buland, R. Constraints on seismic velocities in the Earth from traveltimes. *Geophys. J. Int.* **122(1)**, 108-124 (1995).
3. Simmons, N. A., Myers, S. C., Johannesson, G., & Matzel, E. LLNL−G3Dv3: Global P wave tomography model for improved regional and teleseismic travel time prediction. *J. Geophys. Res. Solid Earth* **117(B10)**, doi:10.1029/2012JB009525 (2012).
4. Zhang, H., & Thurber, C. H. Double-difference tomography: The method and its application to the Hayward fault, California. *Bull. Seis. Soc. Am.* **93(5)**, 1875-1889 (2003).
5. Zhang, H., & Thurber, C. Development and applications of double-difference seismic tomography. *Pure and Applied Geophysics* **163(2)**, 373-403 (2006).
6. Pesicek, J. D., Zhang, H., & Thurber, C. H. Multiscale seismic tomography and earthquake relocation incorporating differential time data: application to the Maule subduction zone, Chile. *Bull. Seism. Soc. Am.* **104(2)**, 1037-1044 (2014).
7. Paige, C. C., & Saunders, M. A. LSQR: An algorithm for sparse linear equations and sparse least squares. *ACM Trans. on Math. Soft.* **8(1)**, 43-71 (1982).
8. Um, J., & Thurber, C. A fast algorithm for two-point seismic ray tracing. *Bull. Seis. Soc. Am.* **77(3)**, 972-986 (1987).
9. Koketsu, K., & Sekine, S. Pseudo-bending method for three-dimensional seismic ray tracing in a spherical earth with discontinuities. *Geophy. J. Int.* **132(2)**, 339-346 (1998).
10. Humphreys, E., & Clayton, R. W. Adaptation of back projection tomography to seismic travel time problems. *J. Geophys. Res. Solid Earth* **93(B2)**, 1073-1085 (1988).

11. Guo, H., McGuire, J. J., & Zhang, H. Imaging the subducted Gorda plate: Implications for the stress state and brittle-ductile transition of the Cascadia subduction zone. *AGU Fall Meeting Abstract* **T13H-0334** (2018).
12. Zelt C. A. Lateral velocity resolution from three-dimensional seismic refraction data, *Geophys. J. Int.* **135**, 1101–1112 (1998).
13. Zhao, D., & Hasegawa, A. P wave tomographic imaging of the crust and upper mantle beneath the Japan Islands. *J. Geophys. Res. Solid Earth* **98(B3)**, 4333-4353 (1993).
14. Hirth, G. & Kohlstedt, D. Rheology of the Upper Mantle and the Mantle Wedge: A View from the Experimentalists. In *Inside the Subduction Factory (*edited by Eiler, J.), *AGU Geophysical Monograph Series* **138**, 83–105 (2003).
15. Mei, S., Suzuki, A.M., Kohlstedt, D.L., Dixon, N.A. & Durham, W.B. Experimental constraints on the strength of the lithospheric mantle. *J. Geophy. Res. Solid Earth* **115(B8)**, doi: 10.1029/2009JB006873 (2010).
16. Stixrude, L. & Lithgow-Bertelloni, C. Thermodynamics of mantle minerals - II. Phase equilibria. *Geophys. J. Int.* **184,** 1180-1213 (2011).
17. Jiang, G., Zhao, D. & Zhang, G. Seismic evidence for a metastable olivine wedge in the subducting Pacific slab under Japan Sea. *Earth Planet. Sci. Lett.* **270**, 300-307 (2008).
18. Frohlich, C. Deep earthquakes. Cambridge University Press, New York, pp 252–301 (2006).
19. Emmerson, B. & McKenzie, D. Thermal structure and seismicity of subducting lithosphere. *Phys. Earth Planet. Inter.* **163**, 191-208 (2007).
